# Using Multisource Feedback to Assess Resident Communication Skills: Adding a New Dimension to Milestone Data

**DOI:** 10.31486/toj.19.0054

**Published:** 2020

**Authors:** Angela Byrd, Kelechi Iheagwara, Pamela McMahon, Michael Bolton, Melissa Roy

**Affiliations:** ^1^ Pediatric Hospital Medicine, Our Lady of the Lake Children's Hospital, Baton Rouge, LA; ^2^ Pediatric Residency Program, Academic Research, Our Lady of the Lake Children's Hospital, Baton Rouge, LA; ^3^ Pediatric Intensive Care Unit, Our Lady of the Lake Children's Hospital, Baton Rouge, LA; ^4^ Pediatric Infectious Disease, Our Lady of the Lake Children's Hospital, Baton Rouge, LA

**Keywords:** *Education−medical*, *pediatrics*

## Abstract

**Background:** The Accreditation Council for Graduate Medical Education (ACGME) requires evaluation of residents’ communication skills. These evaluations should involve assessments from a variety of persons with different perspectives and opportunities to observe resident behavior. Our objectives with this study were to determine if parents, nurses, and physicians significantly differed in their ratings of residents’ communication skills; to ascertain the degree of association between these evaluations and ACGME milestone data; and to elicit feedback from residents about the specificity and usefulness of this type of evaluation compared to the evaluations they were typically provided.

**Methods:** During the 2016-2017 academic year, parents of patients ready for discharge, nurses, and attending physicians completed evaluations of resident communication skills. A repeated measures multivariate analysis of variance compared communication skills scores across the 3 groups of raters. Resident ACGME milestone ratings for interpersonal and communication skills were correlated with the communication skills evaluations. Residents rated the specificity and usefulness of the 360-degree evaluations.

**Results:** Parents rated residents’ communication skills significantly higher than nurses and physicians rated them. We found no significant difference between the nurse and physician ratings. A significant correlation was found between resident ratings by physicians and ACGME milestone data. Residents found the feedback from these evaluations to be more specific and useful in delineating their communication strengths and weaknesses than typical milestone feedback.

**Conclusion:** Parents added a unique perspective about residents’ communication and should be included in resident evaluation when feasible. Residents appreciated the specificity and usefulness of the evaluation instrument.

## INTRODUCTION

The Accreditation Council for Graduate Medical Education (ACGME) requires evaluation of residents’ communication skills.^1^ Faculty have limited time to observe residents and may be overly influenced by their early experiences with the learners.^2^ Evaluation of communication skills should involve multisource assessments from a variety of persons who have had the opportunity to observe the resident in realistic settings because evaluations may differ depending on the rater.^3-6^

Research comparing ratings across provider types has targeted residents in a variety of programs,^4-7^ but we are aware of only 2 research teams in the United States with publications focused on pediatric residents on inpatient ward rotations.^8,9^ Osorio et al investigated whether family-centered rounds, which involve a partnership between the child's family and a multidisciplinary team of providers, represented a good opportunity to assess residents’ interpersonal and communication skills.^8^ Brinkman et al asked parents, nurses, and attending physicians to rate residents on a variety of behaviors indicative of professionalism and communication.^9^ One drawback to the Brinkman et al study is that the researchers used different rating scales and subsets of items across the 3 groups of raters.

Our study also compares resident ratings by parents, nurses, and attending physicians, but we used a consistent set of items and an identical rating scale across the 3 groups of raters. Our objectives were to determine if significant differences among the groups of raters regarding residents’ communication skills existed; to ascertain the degree of association between these evaluations and faculty-generated ACGME milestone data; and to elicit feedback from residents about the specificity and usefulness of this type of evaluation compared to the evaluations they were typically provided.

## METHODS

### Participants

All 32 pediatric residents, postgraduate year (PGY) 1 through PGY 3, on inpatient ward rotations at Our Lady of the Lake Children's Hospital in Baton Rouge, LA from July 2016 through January 2017 were evaluated on their communication skills. English-speaking parents of patients treated by the residents, nursing staff, and attending physicians on our hospitalist service completed evaluations.

### Procedure

The Our Lady of the Lake College, now called Franciscan Missionaries of Our Lady University, institutional review board approved this study as exempt.

A research assistant asked the parents of inpatient pediatric patients ready for discharge from our hospitalist service, which uses family-centered rounds, to evaluate one resident physician who treated their child during the visit. Parents were given an information sheet about the research study and were told that the evaluation was anonymous and voluntary, with no negative repercussions if they declined to evaluate the resident. If parents agreed to participate, they were given the name and a picture of the resident treating their child. Parents were excluded if they did not recognize the resident.

Parents were given a 10-question Parent Evaluation of Resident Survey that Brinkman et al adapted from the internally reliable Patient Satisfaction Questionnaire designed by the American Board of Internal Medicine for adult patients to score their physicians.^9^ Adaptations by Brinkman et al that we also used included changing the word “you” to “your child” for 3 of the 10 items.^9^ Brinkman's team reported that the adapted survey continued to show high internal reliability (α=0.95).^9^ Parents were asked to rate the resident using a 5-point rating scale ranging from poor (1) to excellent (5) and were allowed the option of stating they did not have the opportunity to observe the behavior. Parent demographic information was also collected.

Near the end of the resident's rotation, a research assistant asked ward nurses and attending physicians to complete an anonymous, voluntary questionnaire, rating residents on the same 10 items as the parents. The questionnaire was accompanied by an information sheet explaining that those who choose to respond to the questionnaire were agreeing to participate in a research study. The information sheet also stated that choosing not to respond would have no negative repercussions. Nurses and attending physicians were provided the picture and name of the resident they were asked to evaluate. Response categories included the same 5-point rating scale used by parents, with the option of marking “unable to evaluate” for behaviors not directly monitored during the rotation. Nurses and attending physicians rated more than one resident during the study.

We collected individual resident ACGME milestone ratings for interpersonal and communication skills (ICS) in December 2016. Specifically, we included ICS1 (communicates effectively with patients, families, and the public, as appropriate, across a broad range of socioeconomic and cultural backgrounds) and ICS2 (demonstrates the insight and understanding into emotion and human response to emotion that allows one to appropriately develop and manage human interactions) milestone ratings generated by the residency program's Clinical Competency Committee. ICS ratings are largely based on mean scores given by attending physicians to residents on monthly rotation evaluations. We compared the mean ratings of each resident per rater group to the resident scores on the ACGME ICS1 and ICS2 milestones.

Thirty residents gave consent to allow the research team to analyze their information described above. Two residents declined consent; their data are not included in this report.

The pediatric residency program directors provided residents with information from the multisource evaluation in April 2017. Residents were offered an opportunity to complete a survey on the specificity and usefulness of the multisource evaluation. Rating scales ranged from 1 (not at all [specific]/not at all likely [to make changes]) to 7 (much more [specific]/extremely likely [to make changes]). Open-ended questions about how the evaluations may influence their future communication practices and suggestions to improve the feedback process concerning their communication skills were posed at the end of the anonymous survey. Twenty-nine residents completed the survey.

### Analytic Strategy

We used mean scores for each resident given by each rater group. For example, if 6 parents rated Resident 1, we used the mean parent rating for Resident 1 in our analyses. The mean was calculated based on the number of items with responses.

We conducted a 3 (rater; within group) × 3 (PGY; between group) mixed analysis of variance on the mean rating per resident. To assess the internal consistency of the items, we calculated the Cronbach α coefficient for each group of raters. Pearson correlations were calculated to assess (1) the association between the mean rating given by each rater group and (2) the association between the rater group mean rating and resident ACGME milestone ratings for ICS1 and ICS2. SPSS v. 22.0 (IBM Corp.) was used to conduct these analyses.

Descriptive data from the Resident Follow-Up Survey were calculated, and the research team member leading the data analysis grouped into themes the responses to the open-ended questions concerning what types of changes the residents were likely to implement in their communication with parents and with other healthcare providers in response to the feedback they received. A second researcher analyzed the responses using the generated themes. Disagreements between the 2 researchers about placing responses under themes were flagged, and a third team member made the final determination about how to group the responses.

## RESULTS

### Demographics

Table 1 summarizes demographic characteristics of residents, parents, nurses, and attending physicians. The majority of respondents were female and, depending upon the respondent group, had a mean age between 29 and 36 years. While the majority of residents, nurses, and attending physicians were Caucasian, the majority of parents were African American. The resident group included slightly more first-year (n=11) and slightly fewer third-year (n=9) residents than second-year residents (n=10). Approximately 6% of parents and 21% of nurse responses did not include sex when they completed the survey. Additionally, 7.5% of parents and 21.9% of nurse responses did not indicate race/ethnicity.

**Table 1.** Characteristics of Participants

Characteristic	Residents (n=30)^a^	Parents (n=214)^b^	Nurses (n=137 responses)^b^	Attending Physicians (n=5; 121 responses)^a^
Age, years, mean (SD)	29.4 (2.3)	31.9 (8.1)	30.7 (7.8)	36.1 (2.6)
Sex				
Male	6 (20.0)	14 (6.5)	4 (2.9)	0 (0)
Female	24 (80.0)	187 (87.4)	104 (75.9)	5 (100)
Missing	–	13 (6.1)	29 (21.2)	–
Postgraduate year				
1	11 (36.7)	–	–	–
2	10 (33.3)	–	–	–
3	9 (30.0)	–	–	–
Race/ethnicity				
Caucasian	17 (56.7)	86 (40.2)	83 (60.6)	5 (100)
African American	5 (16.7)	103 (48.1)	20 (14.6)	–
Hispanic	5 (16.7)	6 (2.8)	3 (2.2)	–
Other	3 (10.0)	3 (1.4)	1 (0.7)	–
Missing	–	16 (7.5)	30 (21.9)	–

Note: Data are presented as n (%) unless otherwise indicated.

^a^Demographic information was provided by the residency program.

^b^Demographic responses were collected with each evaluation. Responses from nurses were collected anonymously, so the total number of nurses involved cannot be calculated. This method is the same as that used by Brinkman et al.^9^

### Rating Scale Characteristics

We found a high degree of internal reliability among the 10 items for each group of raters; Cronbach α coefficients ranged from 0.97 for parents and attending physicians to 0.99 for nurses.

Parents and nurses marked <5% of each item “unable to evaluate.” Of 10 total items, attending physicians were able to rate residents 100% of the time on 8 items and 97% of the time on 1 item. However, 33 (27.3%) attending physician surveys were marked as “unable to evaluate” for item 6 (warning children during the physical exam about what he/she is going to do and why; telling the parents what he/she finds).

### Parent, Nurse, and Attending Physician Ratings

An average of 7 parent surveys, 5 nurse surveys, and 4 attending physician surveys were completed per resident. Table 2 presents mean scores for each item on the rating scale across residents by each rating group. Parents rated residents’ overall communication skills the highest ($\bar{X}$=4.73), and attending physicians rated residents’ overall communication skills the lowest ($\bar{X}$=3.91). The mean overall rating given by nurses was 4.08. The difference in communication rating scores across the 3 rater groups was significant (F_(2,54)_=25.765, *P*<0.001). Least significant difference pairwise comparisons demonstrated that parents rated residents significantly higher than nurses (*P*<0.001) and attending physicians (*P*<0.001). The difference between ratings by nurses and attending physicians was not significant (*P*=0.212).

**Table 2.** Mean Rating per Item and Overall for Each Rater Group

HOW IS THIS DOCTOR AT …	Parents	Nurses	Attending Physicians
1. Telling you (parents) everything; being truthful, upfront and frank; not keeping things from you (parents) that you (they) should know	4.75	4.13	4.16
2. Greeting you (parents) warmly; calling you (parents) by the name you (they) prefer; being friendly, never crabby or rude	4.76	4.09	4.19
3. Treating you (parents) like you’re (they’re) on the same level; never “talking down” to you (parents) or treating you (parents) like a child	4.80	4.26	4.07
4. Letting you (parents) tell your (their) story; listening carefully; asking thoughtful questions; not interrupting you (parents) while you’re (they’re) talking	4.76	4.26	4.04
5. Showing interest in you (parents) as a person; not acting bored or ignoring what you (they) have to say	4.77	4.18	4.11
6. Warning your child (children) during the physical exam about what he/she is going to do and why; telling you (parents) what he/she finds	4.72	4.03	3.66
7. Discussing options with you (parents); asking your (parents) opinion; offering choices and letting you (parents) help decide what to do; asking what you (parents) think before telling you (them) what to do	4.64	3.96	3.62
8. Encouraging you (parents) to ask questions; answering them clearly; never avoiding your (their) questions or lecturing you (them)	4.72	4.10	3.97
9. Explaining what you (parents) need to know about your (their) child's problems, how and why they occurred, and what to expect next	4.70	4.13	3.87
10. Using words you (parents) can understand when explaining your (their) child's problems and treatment; explaining any technical medical terms in plain language	4.79	4.11	3.75
Overall mean score	4.73	4.08	3.91

Notes: All data are presented as mean scores by rater group. For all 3 rater groups, the rating scale was as follows: 1=poor; 2=fair; 3=good; 4=very good; 5=excellent.

### Postgraduate Year

PGY level did not influence communication ratings. The difference among communication rating scores given to residents of different PGY levels was nonsignificant (F_(2.27)_=2.523, *P*=0.099).

### Rater × Postgraduate Year Interaction

The interaction between rater and PGY level on communication ratings was significant (F_(4,54)_=3.385, *P*=0.015). Attending physicians rated first-year residents significantly lower than the other two resident years. There was no difference in ratings between resident years for parents and nurses.

### Correlations Between Ratings by Rater Groups

When we compared the association between mean ratings among pairings of the 3 groups, nurse and physician ratings were significantly correlated (r=0.562, *P*=0.01), but parent mean ratings were unrelated to attending physician (r=–0.174) and nurse (r=0.002) mean ratings.

### Correlations Between Ratings and Milestone Data

We found significant correlations between resident ratings by attending physicians and corresponding ACGME milestone data. Specifically, the correlation for milestone ICS1 (communicates effectively with patients, families, and the public, as appropriate, across a broad range of socioeconomic and cultural backgrounds) ratings and mean communication scale ratings given by attending physicians was significant (r=0.7513, *P*<0.001). The milestone rating for ICS2 (demonstrates the insight and understanding into emotion and human response to emotion that allows one to appropriately develop and manage human interactions) and mean communication scale rating given by attending physicians was also significant (r=0.7053, *P*<0.01). These correlations indicate concurrent validity of the communication rating scale for this group of raters. Correlations between the communication rating scale and corresponding milestone data were not significant for parents and nurses.

### Resident Follow-Up Survey

With 7 anchored as “much more” and 1 anchored as “not at all,” residents found the multisource feedback to be more specific ($\bar{X}$=5.88, SD=0.99) and more useful ($\bar{X}$=5.64, SD=1.25) in delineating their communication strengths and weaknesses than the milestone feedback they typically received. With 7 anchored as “extremely likely” and 1 anchored as “not at all likely,” residents also indicated that they were more likely to make changes in how they communicate with parents ($\bar{X}$=5.24, SD=1.45) and with other professionals ($\bar{X}$=5.31, SD=1.41) as a result of the feedback.

In response to an open-ended question concerning changes the residents were likely to make in their communication with parents as a result of the feedback from the multisource evaluation, we identified themes that focused on altering one's demeanor and awareness of behavior, as well as intent to engage in a more active and responsive form of communication. In response to an open-ended question concerning changes the residents were likely to make in their communication with other health professionals as a result of the feedback from the multisource evaluation, we identified themes that focused on speech quality and improving team communication. Four residents responded that they were planning no change in their communication with parents, and 8 residents responded that they were planning no change in their communication with health providers. Table 3 summarizes responses under each question's themes.

**Table 3.** Responses to Resident Follow-Up Survey

Question	Theme	Specific Responses
What kinds of changes are you likely to implement in the ways you communicate with parents?	Altering one's demeanor and awareness	Increase patienceImprove body languageIncrease awareness of presentation/talk during roundsIncrease empathyDon’t interrupt family membersDon’t act shy/deferential when others are in the room (presumably other healthcare providers)Be more sensitive to how new information affects patientsAsk patient's permission or warn them about the exam I am about to do/during physical exams, warn kids about what I am doing and why
	Active and responsive communication	Ask patient permission before doing somethingGive guidance on what to expect during exam/explain what I am doing during examOffer more options/continue to explain therapeutic optionsClearly state physical exam/lab findingsClearly state treatment/discharge planMake sure patient/family understands diseaseBe more transparent when explaining the medical problemAddress family's concernsBe as knowledgeable as possible about the specific caseExplain things more clearly/be more detailedSpeak slower and be more clear when discharging patients
What kinds of changes are you likely to implement make in the ways you communicate with other health professionals?	Speech quality	Speak more loudly/assertivelySpeak more slowlySpeak more calmlyBe graciousDon’t convey fatigue or frustration in tone or attitude
	Improve team communication	Present more conciselyKeep team members updated on plan/changes to plan/orders placedEnsure I am not missing ordersBe more assertiveHelp nursing know why I am not doing something (example, allowing the intern to see something)
	None	None…I am comfortable with who I amNot likely to make changesNone

In response to an open-ended question concerning suggestions on improving the feedback process concerning their communication skills, residents reported that they would like to receive this type of feedback in multiple settings and in real time with examples of their behavior to provide a concrete understanding of which behaviors are problematic.

## DISCUSSION

Our results concur with published reports demonstrating that resident evaluations by parents compared to evaluations by nurses and physicians generated diverse information.^8,10^ Families are likely to observe different aspects of communication involving residents than healthcare professionals. Thus, family evaluations provide residents with a unique perspective regarding how they communicate in the healthcare setting. Therefore, multisource evaluations may warrant the extra time and effort required to collect them.

Osorio et al found that nurses and faculty were more likely to observe particular aspects of resident communication skills after implementation of family-centered rounds.^8^ During family-centered rounds at our facility, 95% of responses given by attending physicians, nurses, and families indicated that they were able to rate residents on 9 of 10 communication behaviors. The exception was that only 72% of physician responses indicated they had the opportunity to observe whether the resident warned children during a physical exam about what they were doing, why they were doing it, and what they found on the exam. In the Brinkman et al study, this item was also the behavior that attending physicians most frequently marked as not observed (59.1% of the time) when using traditional rounds.^9^ Thus, this communication skill remained challenging for attending physicians to observe, even with family-centered rounds.

While the Brinkman et al study contributes important information about the use of multisource evaluations of pediatric residents completing inpatient rotations, the investigators used different rating scales for parents as compared to nurses and attending physicians. Additionally, there were subsets of common items for attending physicians and parents and for attending physicians and nurses, but not for parents and nurses. Thus, a comparison of responses across the 3 groups was challenging.

Brinkman et al concluded that parents and attending physicians were generally alike in their ratings of pediatric residents, but nurses typically rated residents lower than attending physicians. In our study, parents rated residents significantly higher than nurses and attending physicians, but we found no difference between nurse and attending ratings. When ratings are viewed by PGY level and rater group, attending physicians gave PGY 1 residents the lowest average communication score (3.4). Mean ratings for all other combinations of PGY level and rater group were 3.9 or higher. Whether attending physicians have a bias against PGY 1 residents or if they observed aspects of communication behavior that were unique to the observations of parents and nurses is unclear.

Importantly, in our study, attending physician ratings correlated significantly with ACGME milestone data. The milestones are competency-based developmental outcomes used by the ACGME. Since the development of the milestones, residency programs have been challenged with developing accurate and practical ways to assess resident progress using the competencies. In this study, we showed that attending physicians’ assessment of residents using the Evaluation of Resident Survey is an alternative way to assess residents’ communication. The fact that nurse and parent responses did not correlate with attending physician ACGME milestone data raises the question of whether attending physicians’ assessment of resident communication milestones is truly reflective of residents’ communication skills. These data suggest that the different aspects of communication observed by nurses and parents may not be accurately represented in the traditional way we assess resident progress on the communication milestones. This assessment currently involves review of rotation evaluations by physicians along with consensus from the members of the Clinical Competency Committee. Future studies should focus on how much multisource feedback should be included in the communication milestones so that we can continue to improve the accuracy of resident communication skill assessments.

While the collection and analysis of this type of multisource feedback were time consuming, the feedback was perceived as valuable. Residents expressed appreciation for this feedback and rated it as more specific and useful than traditional evaluation data. Future interventions under consideration include incorporating specific responses from Table 3 into monthly rotation orientations; scheduling quarterly resident role-play led by chief residents and faculty during morning reports; and gathering longitudinal data from caregivers to document successful integration into residents’ communication practices.

One limitation of our study is that it was conducted at a single academic medical center with a small sample size. Other studies are needed to assess the generalizability of these results. In addition, resident-peer raters were not included; this group may have opportunities to observe fellow residents in a variety of situations, thus providing a unique perspective.

## CONCLUSION

Multiple raters per group provide stable estimates of various communication skills queried with the reliable and valid evaluation instrument used in this study. Our results suggest that parent evaluations add a unique perspective about residents’ communication and should be included when feasible. Additionally, residents appreciate the specificity and usefulness of the evaluation instrument.

## Notes

This article meets the Accreditation Council for Graduate Medical Education and the American Board of Medical Specialties Maintenance of Certification competencies for Patient Care, Medical Knowledge, and Interpersonal and Communication Skills.
